# Genome-Wide Association Study of Metabolic Syndrome Reveals Primary Genetic Variants at *CETP* Locus in Indians

**DOI:** 10.3390/biom9080321

**Published:** 2019-07-30

**Authors:** Gauri Prasad, Khushdeep Bandesh, Anil K. Giri, Yasmeen Kauser, Prakriti Chanda, Vaisak Parekatt, INDICO, Sandeep Mathur, Sri Venkata Madhu, Pradeep Venkatesh, Anil Bhansali, Raman K. Marwaha, Analabha Basu, Nikhil Tandon, Dwaipayan Bharadwaj

**Affiliations:** 1 Genomics and Molecular Medicine Unit, CSIR-Institute of Genomics and Integrative Biology, New Delhi 110025, India; 2 Academy of Scientific and Innovative Research, CSIR-Institute of Genomics and Integrative Biology Campus, New Delhi 110020, India; 3 Systems Genomics Laboratory, School of Biotechnology, Jawaharlal Nehru University, New Delhi 110067, India; 4 Department of Endocrinology, S.M.S. Medical College, Jaipur, Rajasthan 302004, India; 5 Department of Endocrinology, Centre for Diabetes Endocrinology & Metabolism, University College of Medical Sciences (University of Delhi) & GTB Hospital, New Delhi 110095, India; 6 Dr Rajendra Prasad Centre for Ophthalmic Sciences, All India Institute of Medical Sciences, New Delhi 110029, India; 7 Department of Endocrinology, Post Graduate Institute of Medical Education and Research, Sector-12, Chandigarh 160012, India; 8 Department of Endocrinology, International Life Sciences Institute, New Delhi 110024, India; 9 National Institute of Biomedical Genomics, P.O.: Netaji Subhas Sanatorium, Kalyani 741251, West Bengal, India; 10 Department of Endocrinology, All India Institute of Medical Sciences, New Delhi 110029, India

**Keywords:** genome wide association study, metabolic syndrome, genetic variants, gene regulation

## Abstract

Indians, a rapidly growing population, constitute vast genetic heterogeneity to that of Western population; however they have become a sedentary population in past decades due to rapid urbanization ensuing in the amplified prevalence of metabolic syndrome (MetS). We performed a genome-wide association study (GWAS) of MetS in 10,093 Indian individuals (6617 MetS and 3476 controls) of Indo-European origin, that belong to our previous biorepository of The Indian Diabetes Consortium (INDICO). The study was conducted in two stages—discovery phase (*N* = 2158) and replication phase (*N* = 7935). We discovered two variants within/near the *CETP* gene—rs1800775 and rs3816117—associated with MetS at genome-wide significance level during replication phase in Indians. Additional *CETP* loci rs7205804, rs1532624, rs3764261, rs247617, and rs173539 also cropped up as modest signals in Indians. Haplotype association analysis revealed GCCCAGC as the strongest haplotype within the *CETP* locus constituting all seven *CETP* signals. In combined analysis, we perceived a novel and functionally relevant sub-GWAS significant locus—rs16890462 in the vicinity of *SFRP1* gene. Overlaying gene regulatory data from ENCODE database revealed that single nucleotide polymorphism (SNP) rs16890462 resides in repressive chromatin in human subcutaneous adipose tissue as characterized by the enrichment of H3K27me3 and CTCF marks (repressive gene marks) and diminished H3K36me3 marks (activation gene marks). The variant displayed active DNA methylation marks in adipose tissue, suggesting its likely regulatory activity. Further, the variant also disrupts a potential binding site of a key transcription factor, NRF2, which is known for involvement in obesity and metabolic syndrome.

## 1. Introduction

Metabolic syndrome (MetS) refers to a complex pathophysiological state attained by conjugation of a set of cardiometabolic risk components in an individual that include—central obesity, dyslipidemia, elevated blood pressure, and fasting plasma glucose [1]. MetS aggravates the risk for various health complications including type 2 diabetes (T2D), cardiovascular disease (CVD), cancer, and mortality from all causes [2,3,4]. The prevalence of MetS in India was observed to be ranging from 11–41% on the basis of geographical region, socioeconomic status, urban–rural environment, age, sex, ethnicity of the individuals, and the definition used [5,6,7]. Given the high prevalence of MetS in Indians, with its prompt spread in younger adults [7] and adolescents [8], effective plans for its early detection and intervention are critically desirable to mitigate the burden of associated diseases.

Genetic and environmental factors and their cumulative gene–environment interactions contribute to pathophysiology of MetS [9,10,11,12]. The genetic heritability is liable up to 50% for some individual metabolic components and 13–30% for collective MetS phenotype [9,10,11,12]. Several large-scale genetic studies have been performed to identify MetS related single nucleotide polymorphisms (SNPs) considering independent components of MetS as a quantitative trait [13,14,15,16,17]. In view of MetS as a binary phenotype, several other genome-wide studies worldwide identified numerous loci influencing the combined metabolic syndrome outcome. For instance, genetic loci in *BUD13, ZNF259, APOA5, LPL*, and *CETP* in Europeans [18], *TCF7L2, APOA5, LPL, CETP, APOE*, and *APOC1* in African Americans [19], *CA10* and *CTNNA3* in Africans [20], APOA1/C3/A4/A5 gene cluster region in Finnish [21], *APOA5, BUD13*, and *ALDH2* in Han Chinese [22], *LPL, MYL2, CCDC63*, and *CETP* in Koreans [23], and *APOA* and *COLEC12* in Taiwanese [24] have been attributed for strong association with MetS. These studies highlight multiple shared lipid metabolism pathway genes across diverse populations as well as novel population-specific genes, which require the need for additional population-wide genetic studies to delineate remaining genetic heritability of MetS across varied ethnicities [25].

Indians represent a unique population with a distinct genetic make-up, food habit, and lifestyle compared to other world populations [26,27]. Moreover, Indians display comparatively higher atherogenic dyslipidemia, glucose intolerance, subclinical inflammation, thrombotic propensity, and endothelial abnormality compared to Caucasians [28,29]. Many of such metabolic deregulations are extremely severe and have an earlier age of onset in Indians than Caucasians [28,29]. Besides, Indians have an increased body fat mass, a greater truncal, intra-abdominal subcutaneous adipose tissue with ectopic fat buildup compared to Caucasians, resulting in an enhanced risk for metabolic syndrome and CVD [28,30]. These reasons underscore the possibility of population-specific genetic risk towards MetS phenotype in India. We ourselves have shown this population-specificity of genetic loci for a few metabolic phenotypes in previous reports [15,27], but this is yet unknown for compound MetS phenotype.

Previously, a genome-wide association study was conducted in Indian Asian men (*N* = 4794) of The London Life Sciences Population (LOLIPOP) cohort who were living in West London, United Kingdom (UK) at the time of sample collection [31]. The study lacked a homogenous Indian population as majority of Indian individuals were of mixed ethnicities (Indo-Europeans, Dravidians etc.) in the LOLIPOP cohort that were not appropriately segregated. The study was also limited by only considering Indians living in UK, who differ considerably from native Indians in terms of food habit and lifestyle, which may have an influence on differential genetic architecture for MetS between the two groups. Moreover, the study did not identify genome-wide association study (GWAS) level association (*p* < 5 × 10^−8^) of any genetic variant for compound metabolic syndrome phenotype in representative Indian individuals of London, UK.

The present two-stage genome-wide association study was intended to identify genetic variants governing compound MetS phenotype in 10,093 native Indians (6617 MetS and 3476 controls) speaking Indo-European language.

## 2. Materials and Methods

### 2.1. Ethical Approval

Ethical approval for the study was obtained from Human Ethical Committees of All India Institute of Medical Sciences, New Delhi, India and CSIR-Institute of Genomics and Integrative Biology, New Delhi, India, following principles of Helsinki Declarations (BSC0122, NIDDK GRANT NUMBER: UOO DK085545). All the individuals included in the study were well informed about objectives of study and written consent was taken from each one of them before their involvement in the study.

### 2.2. Study Subjects

All the study subjects were Indo-European speakers from Northern India primarily from neighborhoods of Delhi. In India, genetic ancestry and language are strongly confounded. Moreover, among the Indo-European speakers, the geographical origin of individuals is the essential correlate of genetic variability [32].

The study individuals included in the present study were part of a type 2 diabetes GWAS conducted previously in our laboratory [27] and were members of The Indian Diabetes Consortium (INDICO) [33]. Non-diabetic control subjects who served as controls during GWAS study were enrolled through diabetes alertness camps conducted across different zones of Delhi and adjoining areas [27,33].

Discovery phase T2D subjects were registered from the Department of Endocrinology, All India Institute of Medical Sciences (New Delhi), who joined the clinic before September 2008. Replication phase T2D subjects were registered from departments of collaborator hospitals: All India Institute of Medical Sciences (New Delhi), Guru Teg Bahadur Hospital (New Delhi), and Sawai Man Singh Hospital (Jaipur). T2D patients enrolled from All India Institute of Medical Sciences for replication phase were the patients who joined the clinic post September 2008. In addition, patients with self-known diabetes or under prescription for diabetes and recently diagnosed were also recruited from Diabetes Alertness Camps. T2D subjects were identified as per WHO criteria as described previously [27]. Pregnant females, children, teenagers, and those with type 1 diabetes were excluded from the study.

Blood samples were collected from subjects after an overnight fast, and their DNA was isolated from peripheral blood through salt precipitation protocol. All the study individuals underwent detailed measurements of biochemical and anthropometric measures as described earlier [32]. Waist circumference (WC), fasting glucose (FG), high density lipoprotein cholesterol (HDL-C), triglycerides (TG), and systolic and diastolic blood pressure (SBP and DBP) were measured using standard procedure as described previously [33].

### 2.3. Phenotype Definition (MetS)

We defined MetS using modified National Cholesterol Education Program (NCEP) adult treatment panel (ATP III) measure for Asian populations [34], as used earlier in our previous study [35]. Subjects were classified as MetS cases who attained three or more of these metabolic measures: (1) WC ≥ 90 cm in men or ≥ 80 cm in women, (2) FG ≥ 100 mg/dL or on medication, (3) HDL-C < 40 mg/dL in men or < 50 mg/dL in women or on medication, (4) TG ≥ 150 mg/dL or on medication, and (5) SBP ≥ 130 mm Hg or DBP ≥ 85 mm Hg or taking medication for blood pressure control. Subjects having ≤ 2 number of MetS components were classified as MetS controls.

### 2.4. Genome-Wide Association Study

#### 2.4.1. Discovery Phase

DNA samples were genotyped genome-wide using Illumina Human610-Quad Beadchips (Illumina Inc., San Diego, CA, USA) as part of GWAS studies conducted for T2D and interrelated quantitative metabolic phenotypes earlier in our laboratory [14,15,27,36,37,38]. The GenCall algorithm employed in GenomeStudio software (Illumina, Inc., San Diego, CA, USA) was used to compute genotype calls. The genotype calls were further exported in PLINK v1.07 for downstream analysis [39].

Brief analysis steps and quality control pipeline employed in the study have been summarized in Supplemental Figures S1 and S2. Samples with less than 95% call rate, sex discrepancy, and extremely low or high heterozygosity (mean ± 3 SD) were removed. Further, SNPs with less than 99% call rate, MAF < 0.01, and Hardy Weinberg equilibrium (HWE) *p* < 1 × 10*^−^*^7^ were excluded. Identity by descent (IBD) analysis was performed to identify related and duplicated individuals in the data and those with pi hat score ≥ 0.1875 were excluded. Principal component analysis (PCA) was implemented to spot population outliers. Linkage disequilibrium (LD) pruning of markers was carried in autosomal SNPs using the –indep-pairwise command provided in PLINK v1.07 using r2 of 0.2 and window dimension of 50 SNPs [39]. Analysis of the initial ten principal components detected 41 subjects as potential population outliers (mean ± 6 SD) that were expelled.

Followed by quality control (QC), a total of 519,607 SNPs and 2158 individuals (1596 MetS and 562 controls) remained that were followed for logistic regression analysis assuming an additive model adjusted for age, sex, and the first two principal components in PLINK. Median χ*^2^* statistics were applied to infer genomic inflation factor λ. Manhattan and quantile-quantile (QQ) plots were created using qqman package in R (http://www.r-project.org/) [40].

#### 2.4.2. Replication Phase and Meta-Analysis

The current study is part of a large-scale genetic study to discover genetic variants influencing type 2 diabetes pathophysiology and levels of related quantitative metabolic phenotypes in Indian population [14,15,27,36,37,38]. Markers with discovery phase *p* < 10*^−^*^4^ for MetS and other traits, besides earlier known signals for MetS and other traits, were genotyped in replication phase using GoldenGate assay (Illumina, San Diago, CA, USA). In total, 930 samples (11.72%) were genotyped as technical replicates and an error rate of <0.01% was observed between them.

Samples with less than 90% call rate were removed from the analysis. Further, SNPs with genotype confidence score < 0.25, GenTran score < 0.60, cluster separation score < 0.4 and call rate < 90% were expelled. SNPs with MAF < 0.01 were also discarded. From SNPs with MAF > 0.01, those with HWE *p* < 1 × 10*^−^*^7^ were excluded. After rigorous QC, we retained 2699 SNPs and 7935 individuals (5021 MetS and 2914 controls) in the replication phase that were tested for logistic regression under an additive model adjusted for age and sex.

PLINK [39] was employed for meta-analysis of summary association statistics of discovery and validation phases under a fixed-effect inverse variance model.

Previous associations of identified variants and genes were obtained from the GWAS catalog, GWAS atlas, and Type 2 Diabetes Knowledge Portal [41,42,43]. Regional association plot within ± 1 Mb of lead signal was plotted using locuszoom [44].

### 2.5. Statistical Power of the Study

We calculated the power of the study using Quanto software (Department of Preventive Medicine, University of Southern California, Los Angeles, CA, USA) [45]. Log-additive model of inheritance for allele frequencies in the range from 0.01–0.5 and odds ratios (OR) in the range from 0.63 to 2.08 derived from literature were used. Prevalence of disease was taken as 11% at significance level of 0.05.

### 2.6. Conditional and Haplotype Association Analysis

Conditional analysis for seven variants identified in *CETP* locus was performed in replication phase data employing logistic regression model. Age, sex, and identified SNP genotypes were used as covariates in the model using PLINK.

Haplotype-based association analysis of *CETP* locus was carried out using a logistic regression model adjusting for age and sex at 10,000 permutations in replication phase data using PLINK.

### 2.7. Imputation Analysis

Imputation analysis of novel sub-GWAS loci near *SFRP1* in the discovery phase dataset was performed as detailed earlier [27]. For reference population, 1000 Genome phase 3 panel was used. Prephasing of chromosome 8 was performed with SHAPEIT [46]. A total genomic region of 2 Mb (1 Mb each towards 5′ and 3′ end of the variant) was imputed utilizing IMPUTE 2 [47] which covered the entire LD block of the variant. After imputation, SNPs underwent stringent QC. Imputed SNPs with certainty score <0.90, info score <0.5, and MAF < 0.01 were removed. Further, the QC qualified SNPs were used in association test with compound MetS phenotype using logistic regression adjusting for age, sex, PC1, and PC2 as covariates in the model using PLINK.

### 2.8. Overlaying Gene Regulatory Features

For identification of potential functional relevance of novel sub-GWAS loci near *SFRP1*, we used several publicly available gene regulatory databases. Tissue-wide gene expression profiles of *SFRP1* were downloaded from GTEx-portal-v7 (https://www.gtexportal.org/home/) [48]. ATAC-seq data for human subcutaneous adipose tissue was obtained from an adult female of 53 years of age from ENCODE [49]. ChIP-seq data for regulatory histone marks H3K36me3 or H3K27me3 had been derived from subcutaneous adipose tissue of 5 adult females aged 25, 41, 49, 59, and 81 years, and was obtained from ENCODE [49]. ChIP-seq data for CTCF binding was derived from subcutaneous adipose tissue of 2 adult females aged 51 and 53 years from ENCODE [49]. Whole genome bisulphite sequencing data (WGBS) for adipose tissue was derived from a male adult subject aged 34 years from ENCODE dataset [49]. Predicted sites for transcription factor (TF) binding were obtained from JASPAR portal [50]. UCSC browser was used for visualization of genome regulatory features of sub-GWAS loci [51].

## 3. Results and Discussion

The present study was the first genome-wide association study that identifies a common genetic basis of compound MetS phenotype in Indians of Indo-European origin living in India. Our study was robustly powered to detect loci with similar odds ratios as identified in previous GWAS studies for MetS in literature (>98%) (Supplemental Figure S3). Further, the QQ plot displayed good agreement of calculated *p*-values with theoretical *p*-values under the null hypothesis (Supplemental Figure S4). The genomic inflation factor (λ) was observed to be 1.06 reflecting a homogenous study population. Characteristics of the study population are presented in Supplemental Table S1.

### 3.1. Genome-Wide Association Analysis of MetS

**Figure 1:**
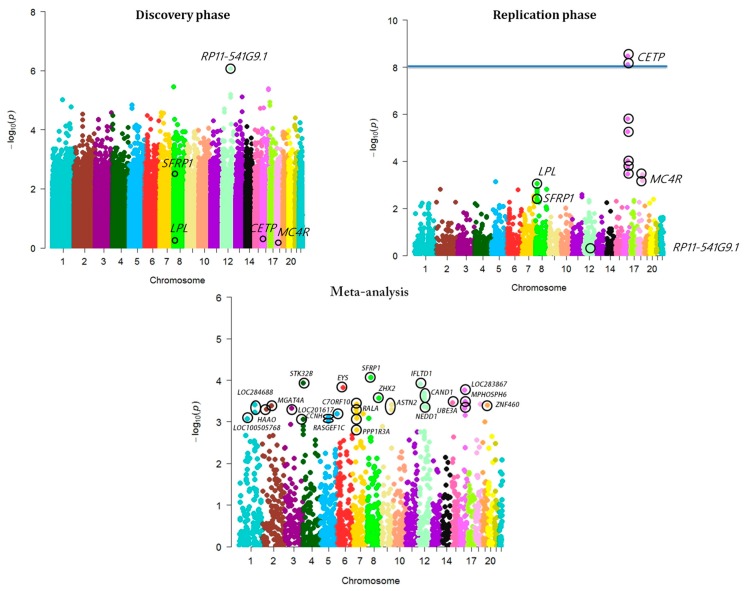
Manhattan plot of association p-values for metabolic syndrome (MetS) in discovery phase, replication phase, and meta-analysis. The −log10 *p*-values of genotyped single nucleotide polymorphisms (SNPs) calculated from association analysis have been presented with respect to SNP positions across autosomes (National Center for Biotechnology Information Build 37).

In the discovery phase, variant rs11108860, which was located within a long intergenic non-coding RNA gene *RP11-541G9.1*, was the strongest signal (*p* = 8.72 × 10*^−^*^7^) [Figure 1]. Though, in the replication phase, association of *RP11-541G9.1* was not sustained (*p* = 0.96).

Interestingly, amid the earlier known gene regions for MetS and related metabolic traits that were genotyped in the replication phase, variants-rs1800775 (*p* = 3.48 × 10*^−^*^9^) and rs3816117 (*p* = 7.71 × 10*^−^*^9^) within/near the *CETP* gene were associated with MetS at genome-wide significance levels in Indians (Table 1, Supplemental Table S2, Figure 1). This was followed by modest associations of an additional five *CETP* loci-rs7205804 (*p* = 1.58 × 10*^−^*^6^), rs1532624 (*p* = 5.57 × 10*^−^*^6^), rs3764261 (*p* = 9.52 × 10*^−^*^5^), rs247617 (*p* = 1.67 × 10*^−^*^4^), and rs173539 (*p* = 3.48 × 10*^−^*^4^) (Table 1, Supplemental Table S2, Figure 1).

The identified GWAS variant near *CETP* in Indians—rs1800775 (C allele)—has been recently demonstrated to have a nominal association with risk of MetS and its individual components (high FG and low HDL-C levels) in the Uyghur ethnic group of China [52]. Besides, another GWAS locus within *CETP*-rs3816117, though not documented in association with MetS until now, is in strong LD with rs1800775 in Africans, Americans, Asians, and Europeans (both r2 and D’ > 0.9) [53], including Indians (r2 = 0.88, D’ = 0.95) [Supplemental Figure S5], suggesting it’s potential association with MetS and related components in certain ethnic origins. Supplemental Figure S6 depicts a regional association plot of signals within/near ± 1 Mb of *CETP* gene region in discovery phase, replication phase and meta-analysis in Indians.

*CETP* gene(cholesteryl ester transfer protein) mRNA is primarily expressed by the spleen, adipose tissue, kidney, liver, lungs, and thyroid [48]. CETP protein plays an important role in the net transport of neutral lipids such as cholesteryl esters and triglycerides [54]. It transfers cholesteryl esters of high-density lipoproteins (HDL) to very low-density lipoproteins (VLDL), in exchange for equimolar amounts of triglyceride from VLDL or chylomicrons to HDL [54]. CETP is also a central protein that maintains the reverse cholesterol transport pathway, wherein overloaded cholesterol is taken from peripheral tissues and restored to the liver for removal from the body [54].

The *CETP* gene is vastly polymorphic. Our identified *CETP* loci serve as strong cis-expression quantitative trait signals (cis-eQTL) in various human tissues including liver stomach, aorta artery, pancreas, and subcutaneous adipose tissue etc., thereby affecting the expression of their occupied gene [Supplemental Table S3]. These variants also confer risk for various cardiometabolic and mental health diseases including dyslipidemia, coronary artery disease, hypertension, obesity, type 2 diabetes, depression, bipolar disorder, and schizophrenia [Supplemental Table S3].

It has been found that high CETP protein activity lowers the concentration of HDL-C [55]. A few functional genetic variants in this gene also present lower plasma protein levels and activity, with parallel increases in HDL-C levels [56,57]. For instance, our identified GWAS locus for MetS—rs1800775-A (protective allele), located in promoter region of the *CETP* gene—has been reported to affect the promoter activity and thereby lowers the gene expression [58], which may have further influence on encoded protein function to maintain lipid levels in the body. This was further demonstrated in another interesting report where subjects with rs1800775-A displayed reduced CETP protein level and activity, and higher HDL-C and apolipoprotein A-I concentrations [59].

On similar lines, rs1800775-A has been shown to confer protection for coronary artery disease (CAD) in a recent large-scale GWAS conducted in individuals from the United Kingdom [60]. In addition to rs1800775, other *CETP* variants, rs247616 and rs1532624, have also been linked to influencing the risk of CAD in Polish populations [61], and rs173539 with coronary artery calcification in a GWAS conducted with Finnish individuals [62].

Further, two variants near *MC4R*-rs17782313 (*p* = 3.66 × 10*^−^*^4^) and rs12970134 (*p* = 6.75 × 10*^−^*^4^), and one variant near *LPL*-rs4128744 (*p* = 8.82 × 10*^−^*^4^) were also modestly associated with MetS in the replication phase in Indians (Table 1, Supplemental Table S2, Figure 1). Discovered *MC4R* locus is a previously documented signal for obesity and type 2 diabetes (rs17782313 and rs12970134) in multiple ethnic cohorts [41]. Moreover, one variant within *ZNF259*-rs964184 (*p* = 2.61 × 10*^−^*^3^), an earlier reported variant for MetS [21], also featured a nominal association with MetS in Indians (Supplemental Table S4).

Genetic variance is essentially governed by multiple SNPs of little effect size that are frequently neglected due to strict GWAS *p*-value limits and multiple testing corrections [63]. We did not detect any genome-wide significant signal in combined analysis during meta-analysis of the discovery and replication phases (Table 2). We only observed a novel sub-GWAS level association (*p* < 1 × 10*^−^*^4^) of locus rs16890462 (*p* = 8.75 × 10*^−^*^5^), that is 23kb 5’ of *SFRP1* locus, for the first time in association with MetS in Indians (Table 2, Figure 1). This was followed by modest associations with other signals in *STK32B, IFLTD1, EYS, CAND1, ZHX2, LOC283867, UBE3A, MPHOSPH6, C7orf10, ASTN2, LOC284688, ZNF460, MGAT4A, NEDD1, LOC201617, HAAO, RALA, PPP1R3A, RASGEF1C, CCNH*, and *LOC100505768* genes (Table 2, Figure 1).

### 3.2. Conditional Analysis of CETP Locus

To classify robust independent variants within the *CETP* locus, conditional analysis was performed using a logistic regression model. Identified GWAS significant *CETP* loci—rs1800775 and rs3816117—were the primary leading signals in the MetS-associated *CETP* region (Supplemental Table S5). Association of other variants with MetS were lost upon adjusting for genotypes of rs1800775 and rs3816117.

However, the remaining five *CETP* loci—rs7205804, rs1532624, rs3764261, rs247617, and rs173539—also had prominent influence on leading GWAS loci (rs1800775 and rs3816117), as adjustment with genotypes of these five loci resulted in loss of genome-wide significance of GWAS loci, though nominal significance was maintained (Supplemental Table S5).

All the identified *CETP* SNPs are in moderate–high LD with each other in Indians (r2 = 0.32–0.99, D’ = 0.75–1) (Supplemental Figure S5).

### 3.3. Haplotype Association Analysis

Haplotype analysis revealed a stronger haplotype within *CETP* locus (OR = 1.26, *p* = 7.97 × 10^−8^ for GCCCAGC haplotype) harboring risk alleles of SNPs rs173539, rs247617, rs3764261, rs1800775, rs3816117, rs7205804, and rs1532624 respectively for association with MetS in Indians (Supplemental Table S6).

### 3.4. SFRP1—A Novel Sub-GWAS Locus for MetS in Indians

We searched for all reported genetic variants within/near our novel sub-GWAS significant *SFRP1* locus. The region has never been reported for metabolic syndrome [43]. However, an intergenic signal-rs973441 near *SFRP1* has been robustly associated (*p* = 3.4 × 10*^−^*^8^) with Type 2 diabetes in a GWAS conducted in Europeans [43]. Indeed, the *SFRP1* locus has also been modestly associated with all MetS component phenotypes including—waist circumference, fasting glucose, HDL cholesterol, triglycerides, and systolic and diastolic blood pressure, as evident from literature [43].

### 3.5. Imputation Analysis of Novel Locus

For *SFRP1*, we perceived few variants that exhibited greater significance with MetS than index variant rs16890462 (Supplemental Table S7). Imputation analysis pipeline has been briefly described in Supplemental Figure S7. A few imputed variants (rs57208963, rs73628732, rs58109926, rs11986767, and rs76305295) were positioned in key regulatory elements and modulated strong binding sites for several key transcription factors like RREB1, Nkx2-5, SREBF2, HNF4G, and EBF1, respectively, that have been already implicated earlier in the context of metabolic disease and associated complications. The identified variants may affect the binding of these transcription factors to *SFRP1* genic regions, and thereby alter the transcriptional and translational levels of *SFRP1* mRNA and protein levels.

### 3.6. SFRP1, a Biologically Relevant Locus

Interestingly, the *SFRP1* gene was found to be considerably expressed in human subcutaneous/visceral adipose tissue and kidney-cortex, and weakly in liver, skeletal muscle, pancreas, and whole blood [Supplemental Figure S8]. This gene encodes SFRP1 protein (secreted frizzled related protein 1), which is a soluble inhibitor of Wnt/β-catenin signaling pathway [54], a key pathway that maintains adipocyte differentiation [64,65]. It has been shown that adding recombinant SFRP1 protein to 3T3-L1 adipocyte cells hampers the antiadipogenic Wnt/β-catenin pathway and stimulates preadipocyte differentiation [66]. Further, another interesting study found *SFRP1* deficient mice to display augmented adiposity, deregulated glucose homeostasis, and elevated inflammation in response to diet induced obesity [67].

In humans, both RNA and protein levels of SFRP1 are increased in slightly obese subjects that get lowered in morbidly obese individuals due to extreme body weight [66]. Moreover, increased obesity results in elevated proinflammatory cytokine secretion and macrophage-infiltration where SFRP1 and Wnt5a are speculated to modulate the inflammatory response. For instance, Wnt5a, which is secreted by antigen presenting cells in joints of rheumatoid arthritis patients, facilitates production of cytokines, like Interleukins (IL-1, IL-6 and IL-8) via Fzd5-CamKII non-canonical Wnt signaling [68]. SFRP1 has been demonstrated to hinder this process [69] and also inhibits the activation of leukocytes and cytokine production in vitro [70], in addition to reducing infiltration of neutrophils in ischemic tissue in vivo [71]. These studies indicate an important role of *SFRP1* in fine tuning the adipogenesis, glucose metabolism and, inflammatory response.

Besides, adipose tissue, *SFRP1* is also considerably expressed in the kidney cortex (Supplemental Figure S8). The Wnt/β-catenin pathway, which is also regulated by SFRP1 protein, is a critical regulator of various important cellular functions including maintenance of homeostatic state, embryonic development, and tissue injury [72]. This pathway also gets activated during kidney development and renal injury besides adipocyte differentiation [64,65,73].

A study found increased SFRP1 protein expression in mice models of kidney injury [74]. In addition, they also found that in these models, kidneys of *SFRP1* knock-out mice showed enhanced renal fibrosis, suggesting it as a protective factor to inhibit renal fibrosis, an initial stage for successive renal diseases [74]. This is in agreement with *SFRP1*’s protective role for influencing a few metabolic syndrome component phenotypes as well [67]. Also, individuals with metabolic syndrome are under greater risk for microalbuminuria/chronic kidney diseases in later stages, depending on the number of aggregated components of metabolic syndrome [75]. In some instances, hypertension, which is one of the components of metabolic syndrome, is considered a prime risk factor for kidney related complications [76]. Thus, metabolic syndrome represents an initial precursor for renal complications where *SFRP1* may serve as an essential protective factor linking metabolic syndrome with renal complications.

**Figure 2:**
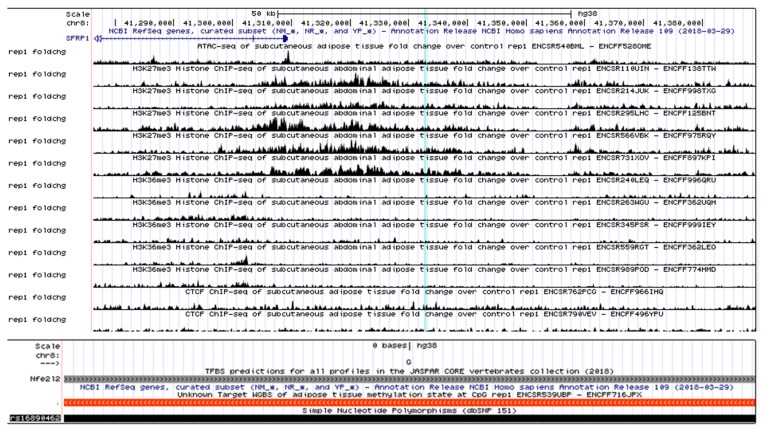
Regulatory features of *SFRP1* locus - rs16890462. Gene regulatory signatures in human subcutaneous adipose tissue. H3K36me3: Active transcription; H3K27me3: Repressed transcription; ATAC-seq peaks: Open chromatin; CTCF: TF that is enriched in repressed genic regions [ENCODE data]. Predicted binding sites for TFs where gray scale denotes enrichment of TF [JASPAR data].

Further, to decode the likely functional role of our identified *SFRP1* variant, rs16890462, we explored the open chromatin features, active and repressive histone modifications (H3K36me3 and H3K27me3 respectively), transcription factor (TF), and CTCF binding sites in human subcutaneous adipose tissue. ATAC-seq and histone marks data suggested the desired region under closed chromatin state in subcutaneous adipose tissue, displayed by substantially higher enrichment for H3K27me3 and CTCF marks and an absence of H3K36me3 marks, which symbolizes repressive chromatin state (Figure 2). Further, dynamic peaks of CTCF binding and hypermethylation (from WGBS data) at variant regions in adipose tissue indicates its potential regulatory activity [Figure 2]. Intriguingly, the variant represented strong binding sites for Nfe212 (also known as NRF2), a crucial transcription factor implicated in obesity and metabolic syndrome [77].

## 4. Conclusions

Lipid metabolism is closely coupled with energy balance cycle and physiological homeostasis. Lipid molecules are involved in diverse biological functions ranging from acting as long-term energy depots to a variety of signaling functions. Deregulated lipid metabolism is increasingly being implicated in plentiful metabolic diseases including obesity, cardiovascular disease, and diabetes. *CETP* as a quantitative trait locus for lipid levels in human body is already established. Here, our study reveals association of *CETP* loci with MetS, which is a precursor for numerous complex disease phenotypes. So, in future, *CETP* may serve as a potential drug target for MetS.

In conclusion, our study assigns *CETP*, a known gene controlling lipid homeostasis, as a major locus for regulating MetS pathophysiology in Indians. We also discovered a novel sub-GWAS locus in *SFRP1*, which has already been functionally tested in mice and humans to regulate a few individual components of MetS, including obesity and glucose metabolism.

## Figures and Tables

**Table 1:** Association status of known metabolic traits associated gene regions with MetS in replication phase in Indians (*p* < 1 × 10^−3^).

Replication Phase					
Gene Region	CHR	Start Base Pair (hg19)	Number of SNPs	*p*-Value (Lead SNP)	N
*CETP*	16	56988044	7	3.48 × 10^−9^	4671
*MC4R*	18	57851097	2	3.66 × 10^−4^	4666
*LPL*	8	19919655	1	8.82 × 10^−4^	4650

Association analysis with compound MetS phenotype, adjusted for age and sex as covariates. Start base pair is position of farthest 5′ SNP in context of gene. CHR: Chromosome; N: Number of non-missing individuals.

**Table 2:** Novel signals associated with MetS (*p*-value < 1 × 10^−3^) in meta-analysis in Indians.

							Discovery Phase			Replication Phase			Meta-Analysis				
SNP	CHR	Base Position	Nearby Gene	SNP Location	Alleles (Effect/Other)	MAF	N	*p*-Value	OR	N	*p*-Value	OR	*p*-Value	OR	Dir	I	Q
rs16890462	8	41309355	*SFRP1*	Intergenic	A/G	0.21	2156	5.48 × 10^−3^	1.29	3259	5.51 × 10^−3^	1.25	8.75 × 10^−5^	1.26	++	0	0.78
rs1530611	4	5206254	*STK32B*	intronic	A/G	0.29	2148	6.69 × 10^−3^	1.23	3257	6.37 × 10^−3^	1.21	1.18 × 10^−4^	1.22	++	0	0.89
rs11048180	12	25735148	*IFLTD1*	intergenic	A/G	0.08	2157	5.46 × 10^−4^	0.66	3259	0.04	0.8	1.25 × 10^−4^	0.73	--	31.56	0.23
rs16896746	6	66289412	*EYS*	intronic	G/A	0.08	2156	4.32 × 10^−5^	0.62	3259	0.1	0.86	1.51 × 10^−4^	0.73	--	75.4	0.04
rs710630	12	65983583	*CAND1*	intronic	G/A	0.46	2158	0.01	1.18	3259	4.61 × 10^−3^	1.19	2.08 × 10^−4^	1.19	++	0	0.91
rs710628	12	65943747	*CAND1*	intergenic	A/G	0.46	2157	0.01	1.18	3258	5.53 × 10^−3^	1.19	2.47 × 10^−4^	1.18	++	0	0.93
rs7005211	8	123538147	*ZHX2*	intergenic	G/A	0.47	2155	6.69 × 10^−3^	0.83	4654	8.90 × 10^−3^	0.89	2.72 × 10^−4^	0.87	--	0	0.33
rs1060350	12	65992732	*CAND1*	synonymous	G/A	0.48	2157	6.15 × 10^−3^	1.21	3254	0.01	1.16	2.85 × 10^−4^	1.18	++	0	0.67
rs1152877	12	65989452	*CAND1*	intronic	G/A	0.48	2155	5.50 × 10^−3^	1.22	3220	0.01	1.16	2.89 × 10^−4^	1.18	++	0	0.62
rs564210	16	64314818	*LOC283867*	intergenic	A/G	0.26	2156	3.55 × 10^−4^	0.76	3251	0.1	0.89	3.30 × 10^−4^	0.83	--	60.29	0.11
rs12595506	15	25744981	*UBE3A*	intergenic	G/A	0.39	2156	0.01	1.19	7930	4.99 ×10^−3^	1.1	3.53 × 10^−4^	1.11	++	0.22	0.32
rs2967379	16	80770811	*MPHOSPH6*	intergenic	G/A	0.49	2156	4.52 × 10^−6^	0.73	3258	0.4	0.96	3.69 × 10^−4^	0.84	--	88.69	2 × 10^−3^
rs10499618	7	40787165	*C7orf10*	intronic	G/A	0.11	2158	3.98 × 10^−3^	1.41	3257	0.03	1.26	3.82 × 10^−4^	1.32	++	0	0.48
rs1337212	9	119239170	*ASTN2*	intergenic	A/G	0.11	2157	4.53 × 10^−4^	1.55	3259	0.09	1.18	3.93 × 10^−4^	1.31	++	62.74	0.1
rs7554931	1	170365623	*LOC284688*	intergenic	A/G	0.41	2157	2.91 × 10^−3^	0.81	4673	0.01	0.9	3.98 × 10^−4^	0.87	--	44.4	0.18
rs3746228	19	57804362	*ZNF460*	3’-UTR	A/G	0.18	2158	7.18 × 10^−4^	1.38	4529	0.03	1.13	4.02 × 10^−4^	1.18	++	70.48	0.06
rs885036	2	98671225	*MGAT4A*	intronic	G/A	0.49	2157	2.96 × 10^−5^	1.35	3254	0.3	1.07	4.18 × 10^−4^	1.18	++	83.49	0.01
rs1066396	12	66005634	*CAND1*	intergenic	G/A	0.48	2157	6.14 × 10^−3^	1.21	3254	0.02	1.15	4.37 × 10^−4^	1.17	++	0	0.59
rs11108860	12	96081536	*NEDD1*	intergenic	G/A	0.04	2157	8.72 × 10^−7^	0.45	3259	0.9	1.01	4.52 × 10^−4^	0.66	-+	91.59	6 × 10^−4^
rs4677119	3	72291958	*LOC201617*	intergenic	A/G	0.33	2158	5.76 × 10^−3^	0.81	4653	0.01	0.89	4.80 × 10^−4^	0.87	--	22.01	0.26
rs9309089	2	43028132	*HAAO*	intergenic	A/G	0.35	2158	0.1	1.12	4623	1.54 × 10^−3^	1.15	5 × 10^−4^	1.14	++	0	0.72
rs6948816	7	39661439	*RALA*	intronic	A/G	0.03	2158	5.36 × 10^−3^	0.62	3257	0.03	0.69	5.13 × 10^−4^	0.65	--	0	0.62
rs10983653	9	119237233	*ASTN2*	intergenic	A/G	0.11	2158	6.15 × 10^−4^	1.53	3259	0.09	1.19	5.14 × 10^−4^	1.31	++	60.43	0.11
rs1333144	1	170364401	*LOC284688*	intergenic	G/A	0.41	2157	5.40 × 10^−3^	0.82	4654	0.02	0.9	5.92 × 10^−4^	0.88	--	27.92	0.24
rs17530234	7	40783104	*C7orf10*	intronic	G/A	0.11	2155	5.49 × 10^−3^	1.38	3258	0.03	1.24	6.25 × 10^−4^	1.3	++	0	0.49
rs2462683	7	112971889	*PPP1R3A*	intergenic	G/A	0.34	2158	6.34 × 10^−4^	1.3	3257	0.1	1.11	6.51 × 10^−4^	1.18	++	59.06	0.12
rs11749727	5	179540965	*RASGEF1C*	intronic	G/A	0.44	2156	6.75 × 10^−3^	1.21	3258	0.03	1.15	6.59 × 10^−4^	1.17	++	0	0.55
rs475479	16	64324819	*LOC283867*	intergenic	G/A	0.26	2158	5.41 × 10^−4^	0.76	3259	0.1	0.9	7.12 × 10^−4^	0.83	--	61.71	0.12
rs35814902	5	86835416	*CCNH*	intergenic	A/G	0.32	2158	5.04 × 10^−3^	1.24	4573	0.02	1.11	8.01 × 10^−4^	1.13	++	38.95	0.2
rs17529882	7	40761636	*C7orf10*	intronic	G/A	0.09	2157	2.10 × 10^−3^	1.48	3259	0.08	1.22	8.28 × 10^−4^	1.32	++	26.33	0.24
rs17456070	1	87599332	*LOC100505768*	intronic	G/A	0.30	2155	0.05	0.86	3257	5.98 ×10^−3^	0.83	8.56 × 10^−4^	0.84	--	0	0.74
rs12650617	4	5238437	*STK32B*	intronic	A/G	0.22	2157	3.35 × 10^−5^	1.45	3245	0.3	1.07	8.81 × 10^−4^	1.21	++	85.49	8.70 × 10^−3^
rs13177543	5	86842168	*CCNH*	intergenic	A/G	0.32	2158	5.04 × 10^−3^	1.24	4658	0.03	1.1	9.93 × 10^−4^	1.13	++	42.89	0.18

Novel loci associated with compound MetS phenotype in meta-analysis, adjusted for age, sex, and first two principal components in discovery phase, and age and sex in replication phase. Meta-analysis was done using fixed effect inverse variance method in PLINK. SNP location is position of SNP in context of gene. CHR: Chromosome; MAF: Minor allele frequency; N: Sample number; OR: Odds ratio; Dir: Direction; I: I2 heterogeneity index (0–100); Q: *p*-value for Cochrane’s Q statistic. Direction ++/-- features a concordance between the discovery and replication phase.

## Supplementary Materials

The following are available online. Figure S1: Brief data analysis pipeline employed in the study; Figure S2: Data quality control executed in discovery and replication phase of the study; Figure S3: Statistical power of the study; Figure S4: Quantile-Quantile plot (QQ plot) between calculated and theoretical distribution of *p*-values in discovery phase; Figure S5: Pairwise linkage disequilibrium (LD) between the 7 *CETP* variants associated with MetS in the present study; Figure S6: Regional association plots of *CETP* signals in discovery phase, replication phase, and meta-analysis; Figure S7: Imputation flow chart for signal near *SFRP1*; Figure S8: Gene expression summary of *SFRP1* in major human tissues related to MetS; Table S1: Characteristics of study population; Table S2: Association status of known metabolic traits associated GWAS variants with MetS in replication phase in Indians (*p* < 1 × 10*^−^*^3^); Table S3: Reported eQTL and trait/disease associations of *CETP* region; Table S4: Association status of known metabolic traits associated GWAS variants with MetS in replication phase in Indians (1 × 10*^−^*^3^ < *p* > 0.05); Table S5: Conditional analysis of *CETP* variants in Indians; Table S6: Haplotype association analysis of *CETP* locus for rs173539, rs247617, rs3764261, rs1800775, rs3816117, rs7205804, and rs1532624 respectively; Table S7: Association analysis of novel imputed *SFRP1* variants with MetS in Indians.

## Members of INDICO

Viswanathan Mohan, Saurabh Ghosh, Abhay Sharma, Rubina Tabassum, Ganesh Chauhan, Anubha Mahajan, Om Prakash Dwivedi, Lakshmi Ramakrishnan, Radha Venkatesan, M. Chidambaram, D. Prabhakaran, K.S Reddy, Monisha Banerjee, Madhukar Saxena, Sandeep Mathur, Anil Bhansali, Viral Shah, S.V. Madhu, R.K. Marwaha, Pradeep Venkatesh, S.K. Aggarwal, Shantanu SenGupta, Sreenivas Chavali, Amitabh Sharma, Analabha Basu, Khushdeep Bandesh, Anil K Giri, Shraddha Chakraborty, Yasmeen Kauser, B. Abitha, Aditya Undru, Donaka Rajashekar, Gauri Prasad, Punam Jha, Priyanka Banerjee, Vaisak Parekatt, Suki Roy, Anjali Singh, Nikhil Tandon and Dwaipayan Bharadwaj (Coordinator).
