# Supplementary material for: Genome-Wide Association Study of Metabolic Syndrome Reveals Primary Genetic Variants at CETP Locus in Indians
Source: Biomolecules. 2019 Jul 30;9(8):321. doi: 10.3390/biom9080321 (PMC6723498; doi:10.3390/biom9080321)
Supplement: Supplementary file 1 [file biomolecules-09-00321-s001.pdf]

Figure S1: Brief data analysis pipeline employed in the study

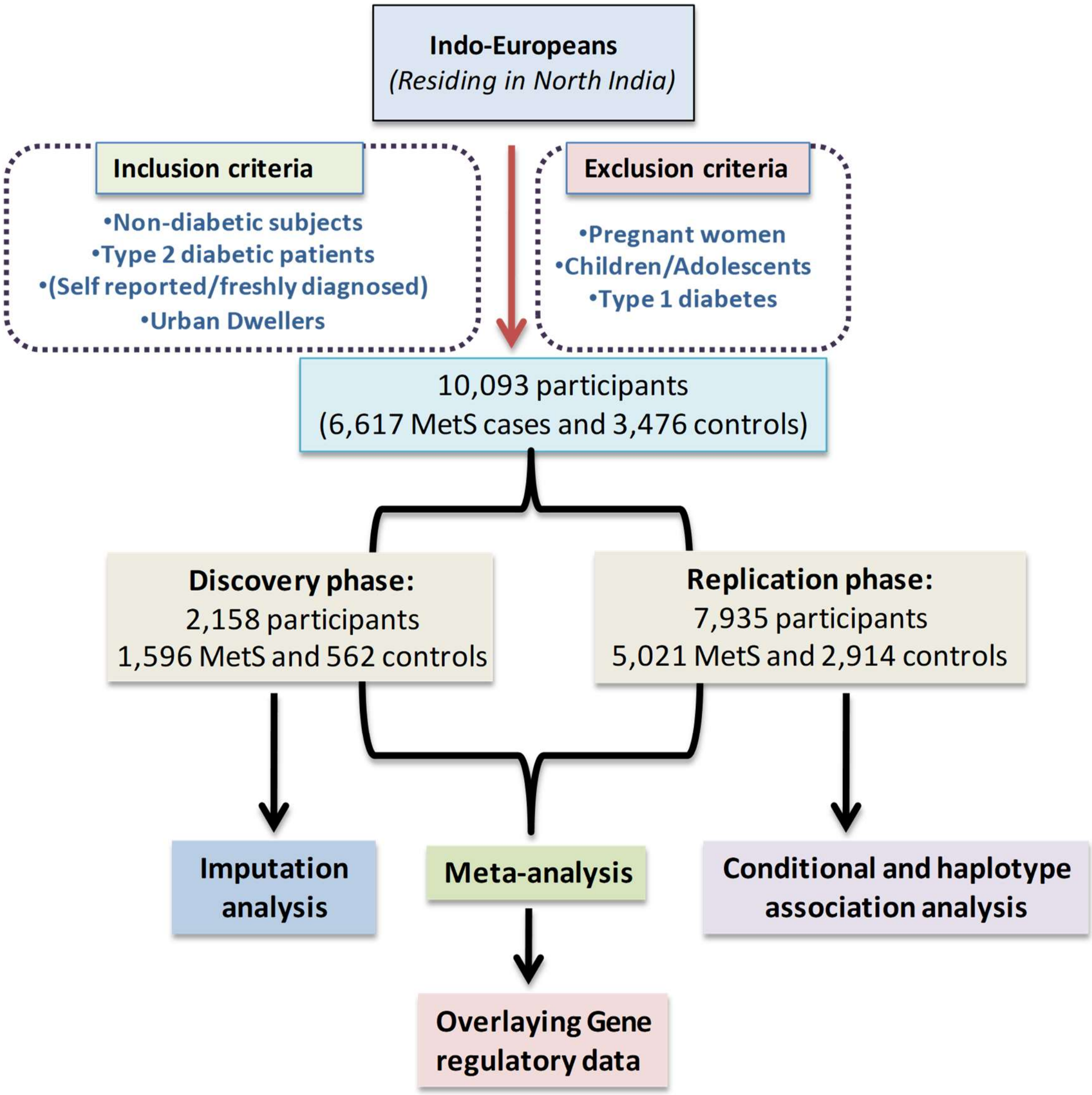

Figure S2: Data quality control executed in discovery and replication phase of the study

(a)

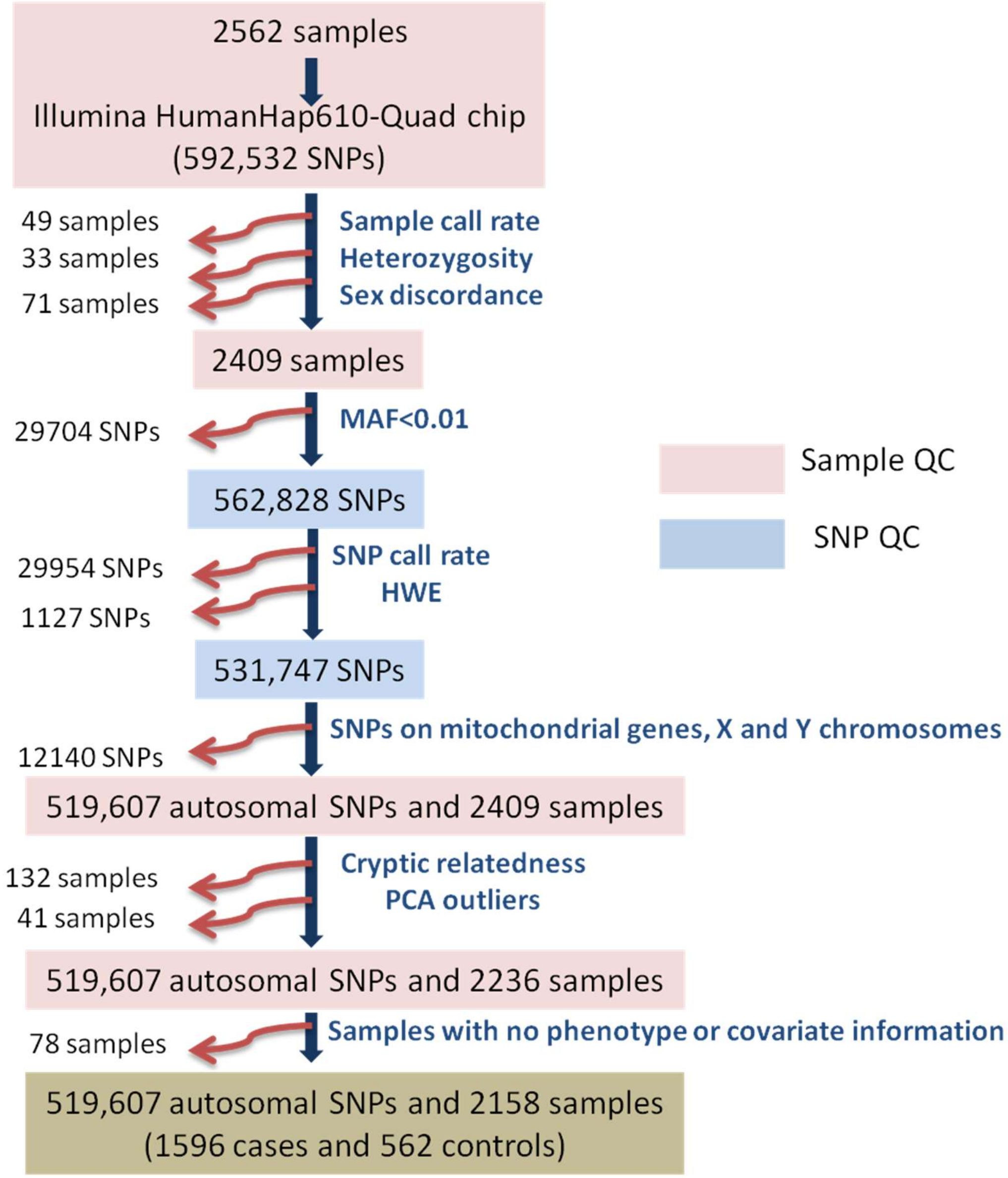

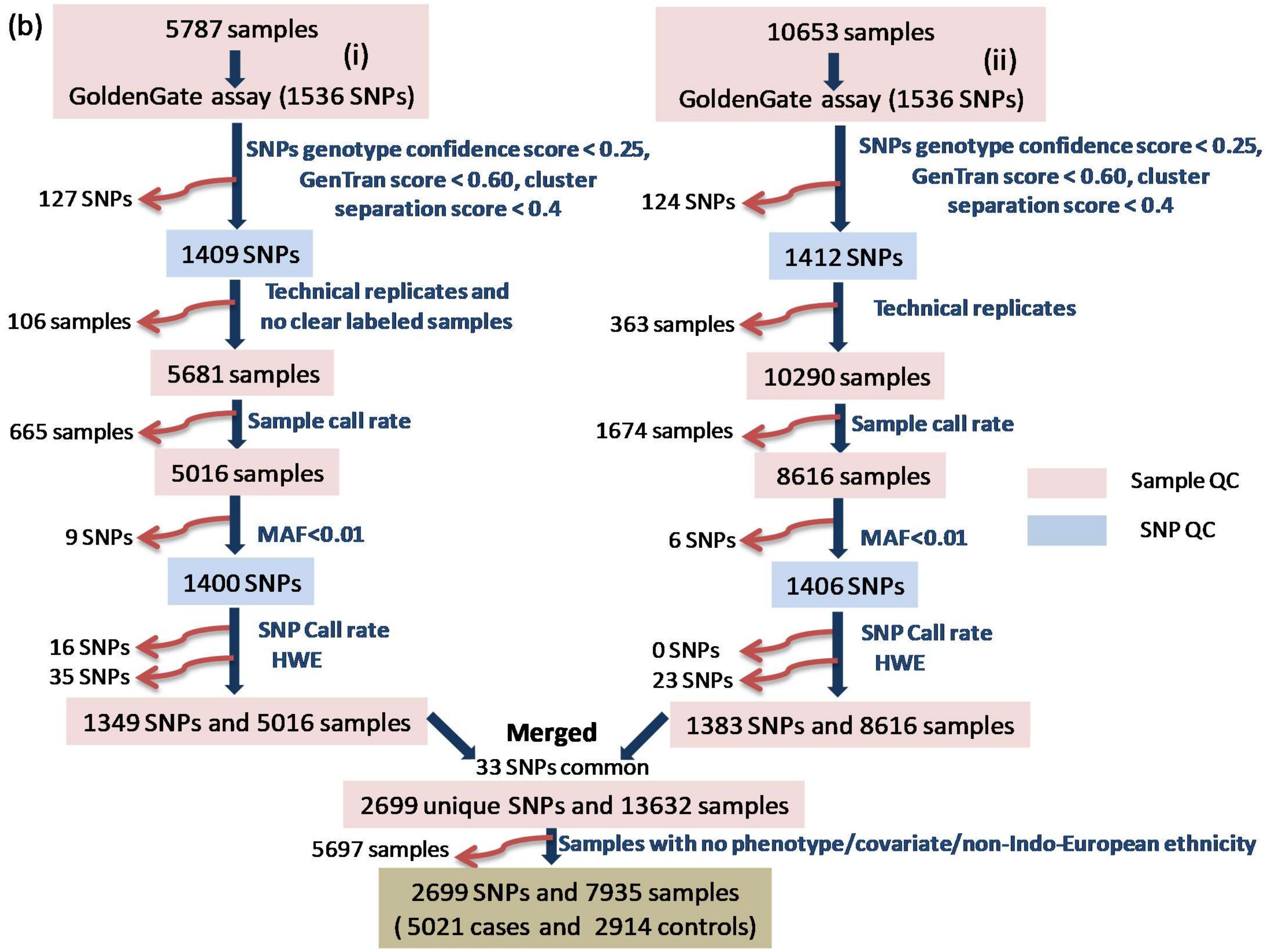
(b)
5787 samples
(i)
GoldenGate assay (1536 SNPs)
SNPs genotype confidence score < 0.25, GenTran score < 0.60, cluster separation score < 0.4
127 SNPs
1409 SNPs
Technical replicates and no clear labeled samples
106 samples
5681 samples
Sample call rate
665 samples
5016 samples
MAF<0.01
9 SNPs
1400 SNPs
SNP Call rate
HWE
16 SNPs
35 SNPs
1349 SNPs and 5016 samples
10653 samples
(ii)
GoldenGate assay (1536 SNPs)
SNPs genotype confidence score < 0.25, GenTran score < 0.60, cluster separation score < 0.4
124 SNPs
1412 SNPs
Technical replicates
363 samples
10290 samples
Sample call rate
1674 samples
8616 samples
MAF<0.01
6 SNPs
1406 SNPs
SNP Call rate
HWE
0 SNPs
23 SNPs
1383 SNPs and 8616 samples
Sample QC
SNP QC
Merged
33 SNPs common
2699 unique SNPs and 13632 samples
Samples with no phenotype/covariate/non-Indo-European ethnicity
5697 samples
2699 SNPs and 7935 samples
(5021 cases and 2914 controls)

Analysis quality control executed in (a) discovery phase (b) replication phase of the study: (i) Independent replication phase was carried for follow-up of variants moderately associated ($p<10^{-4}$) with quantitative metabolic traits, metabolic syndrome (ii) and, Type 2 diabetes in Indians

Figure S3: Statistical power of the study

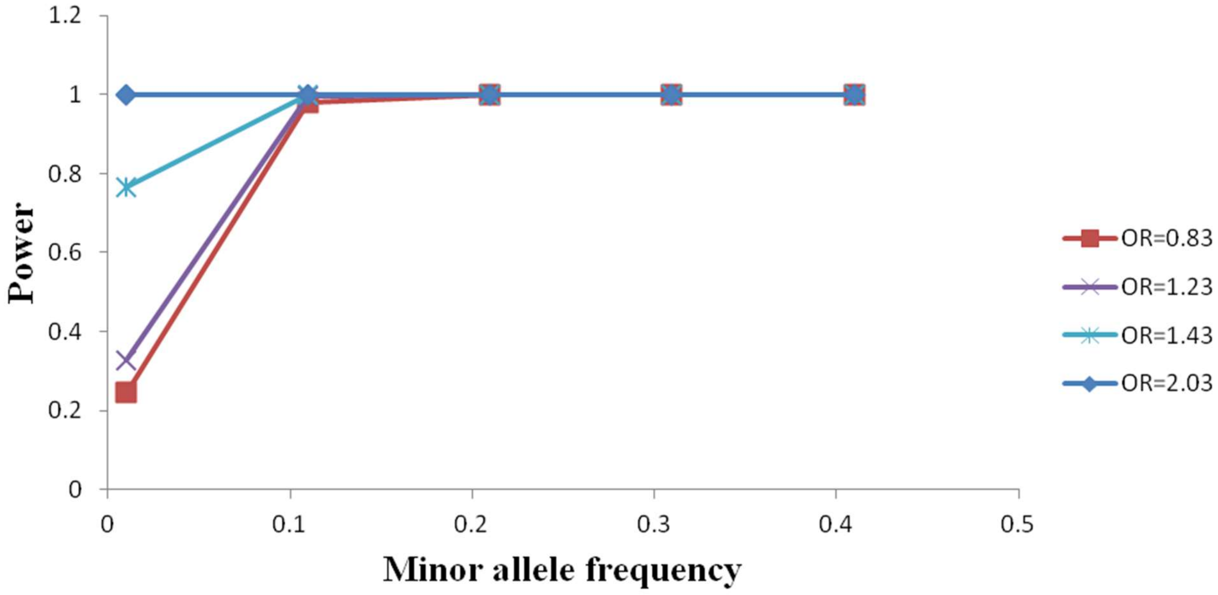

The power of the study for meta-analysis was computed for allele frequencies in the range from 0.01 to 0.50 for odds ratio ranging from 0.63-2.08 using log-additive model of inheritance at p-value = 0.05

Figure S4: Quantile-Quantile plot (QQ plot) between calculated and theoratical distribution of p-values in discovery phase

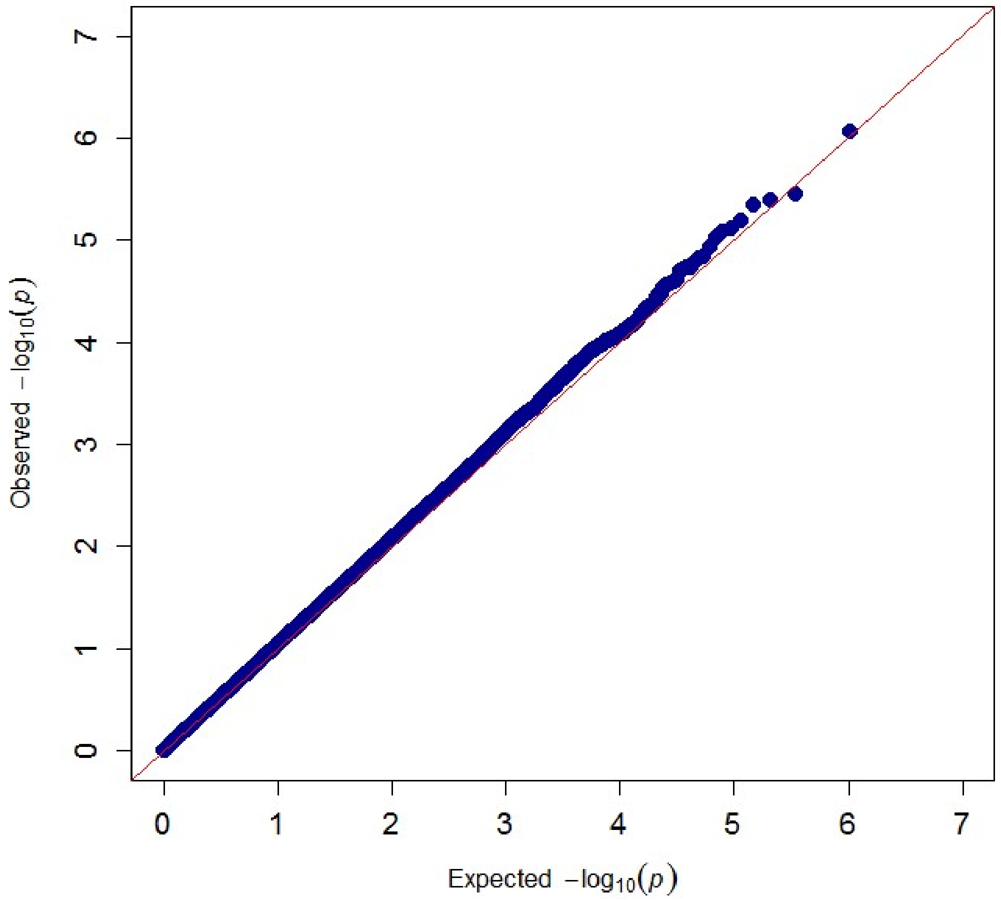

Quantile-quantile (QQ) plot for the observed p-values in discovery phase. The -log10 of observed p-values observed with association for MetS assuming additive model adjusted for age, sex, PC1 and PC2 (blue symbols) have been drawn against the theoretical -log10 p-values that are expected under the null hypothesis (red line). The genomic control inflation factor (λ) was calculated to be 1.06

Figure S5: Pairwise linkage disequilibrium (LD) between the 7 *CETP* variants associated with MetS in the present study

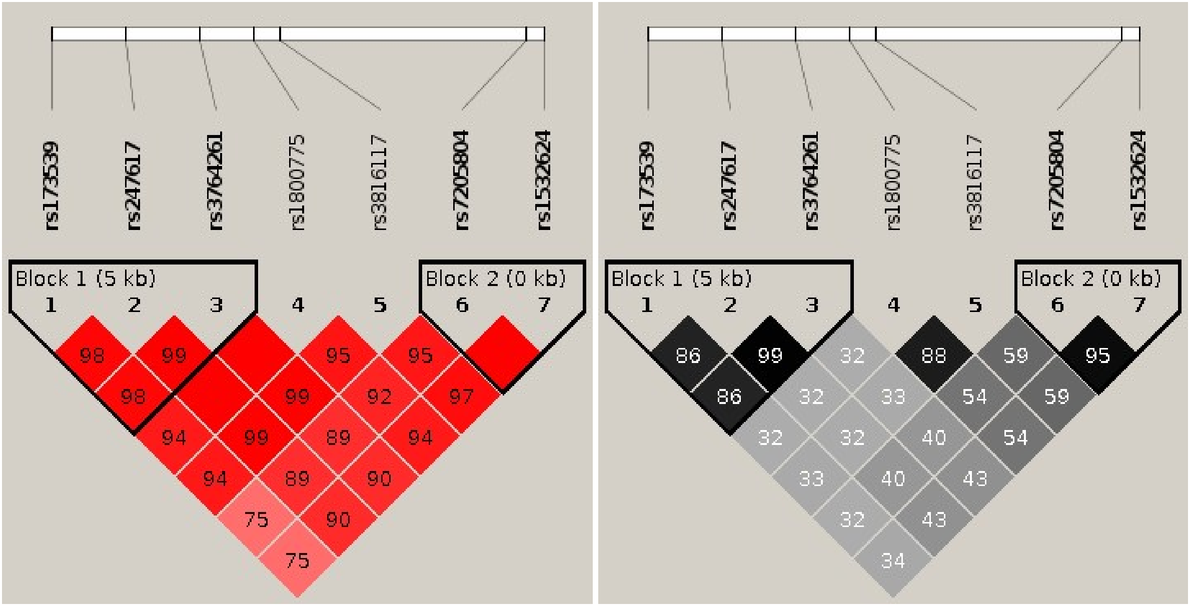

Left panel represents the LD plot with D' values and right panel represents the LD plot with r2values.

Figure S6: Regional association plots of *CETP* signals in discovery phase, replication phase and meta-analysis

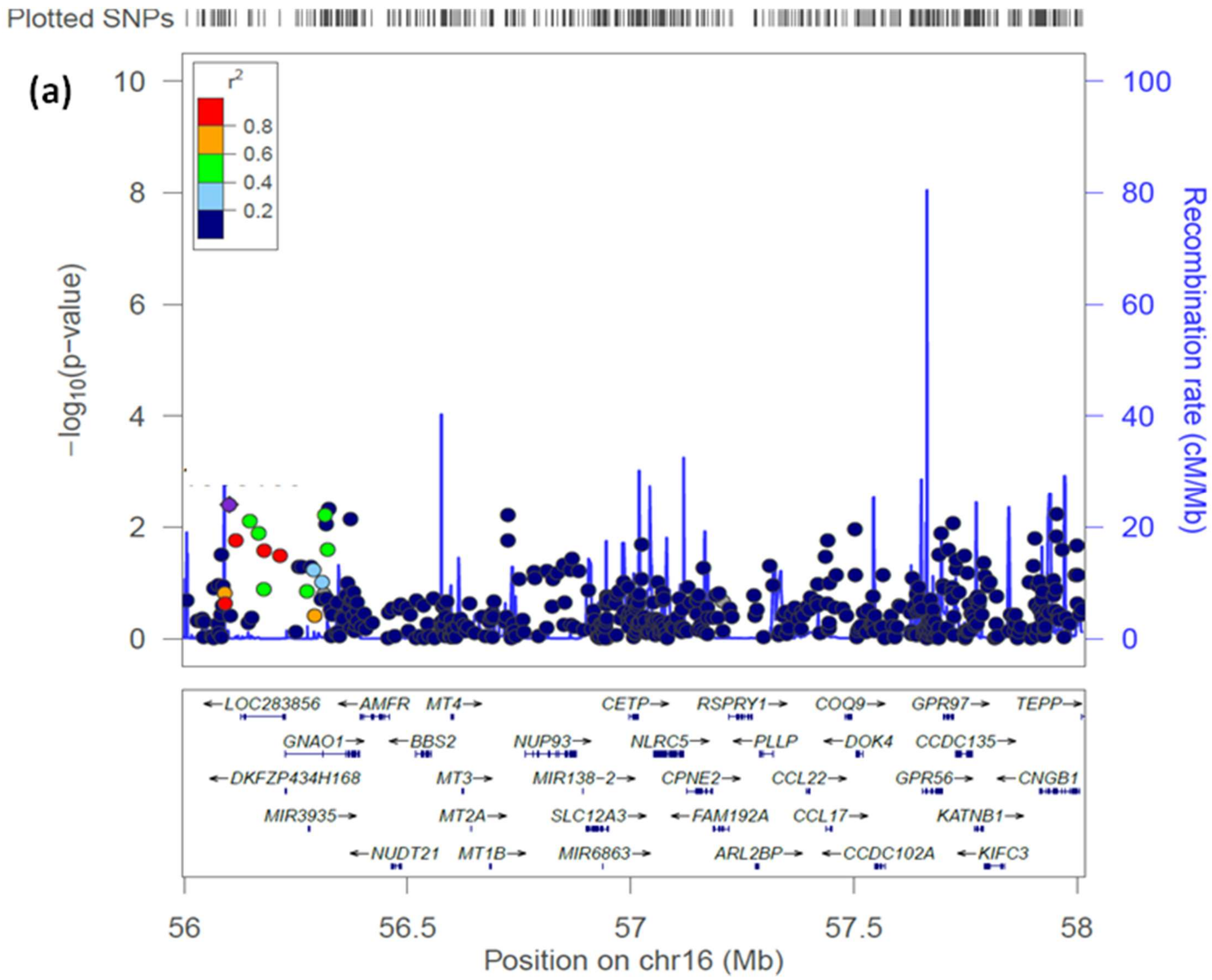

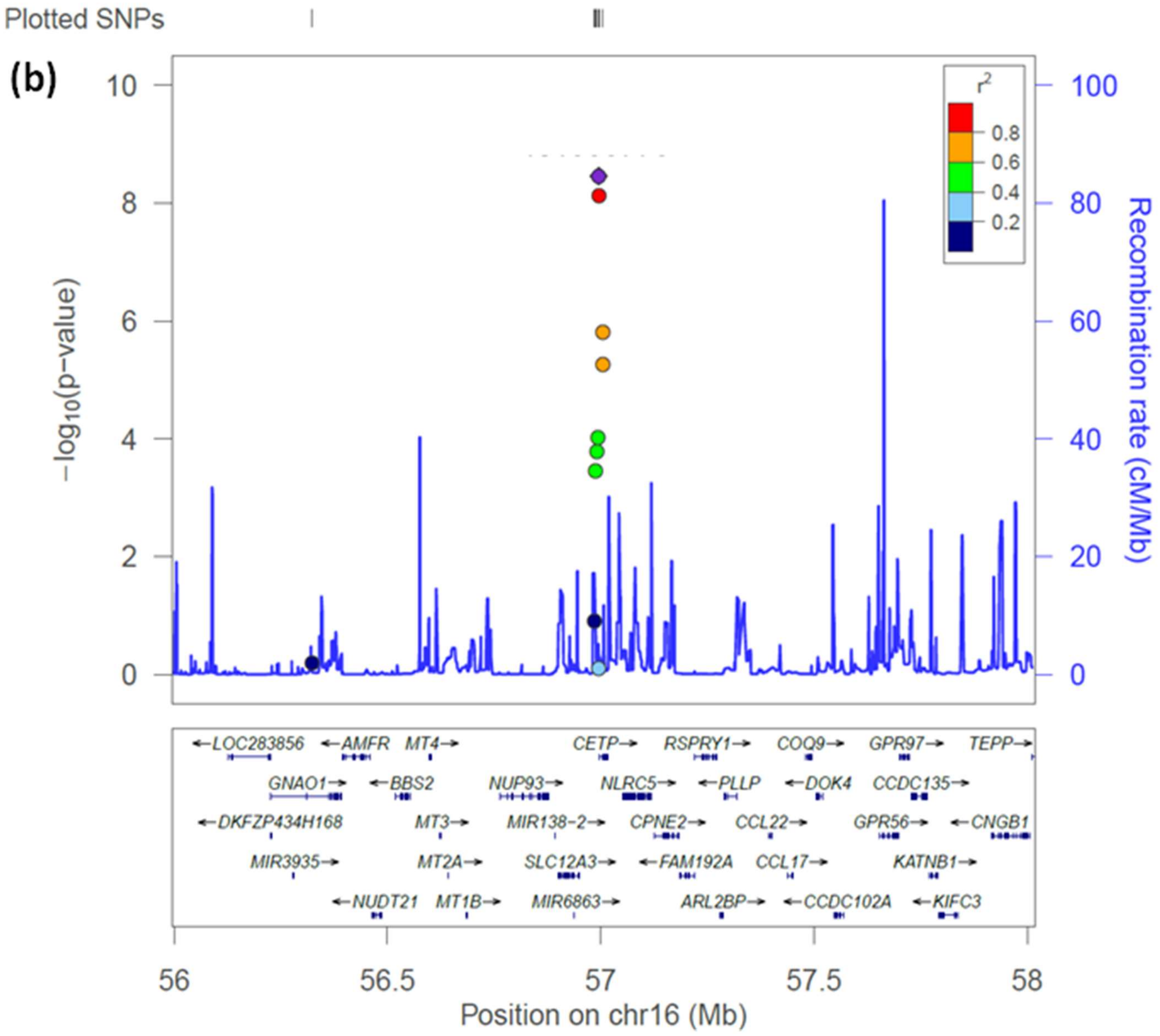
Plotted SNPs
(b)
−log10(p−value)
Recombination rate (cM/Mb)
r2
0.8
0.6
0.4
0.2
LOC283856
AMFR
MT4
CETP
RSPRY1
COQ9
GPR97
TEPP
GNAO1
BBS2
NUP93
NLRC5
PLLP
DOK4
CCDC135
DKFZP434H168
MT3
MIR138-2
CPNE2
CCL22
GPR56
CNGB1
MIR3935
MT2A
SLC12A3
FAM192A
CCL17
KATNB1
NUDT21
MT1B
MIR6863
ARL2BP
CCDC102A
KIFC3
Position on chr16 (Mb)

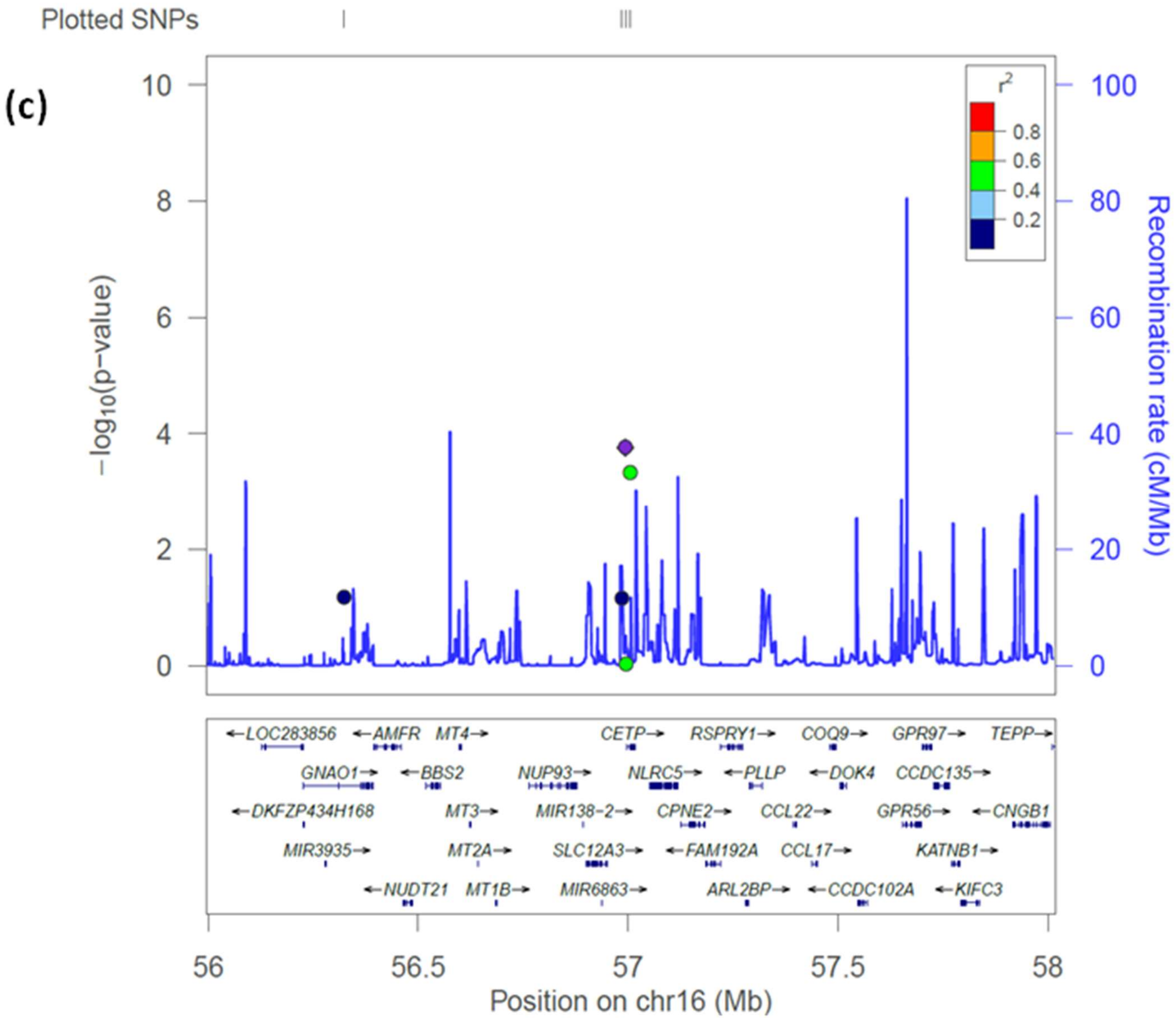

Regional association plots around ± 1 Mb of *CETP* region in (a) discovery phase, (b) replication phase and, (c) meta-analysis. Regional association plot was created using locuszoom (http://locuszoom.org/).

Figure S7: Imputation flow chart for signal near *SFRP1*

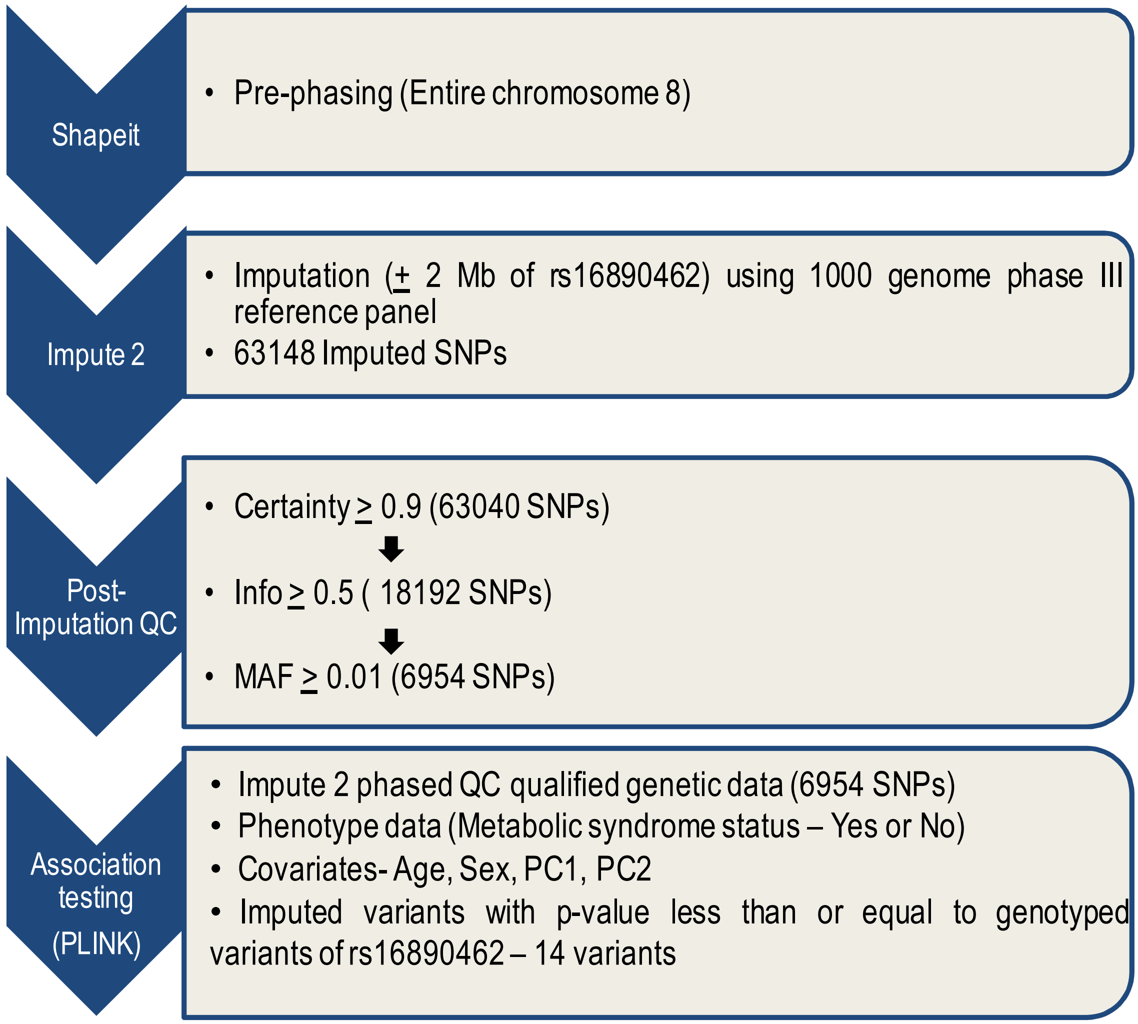

Figure S8: Gene expression summary of *SFRP1* in major human tissues related to MetS

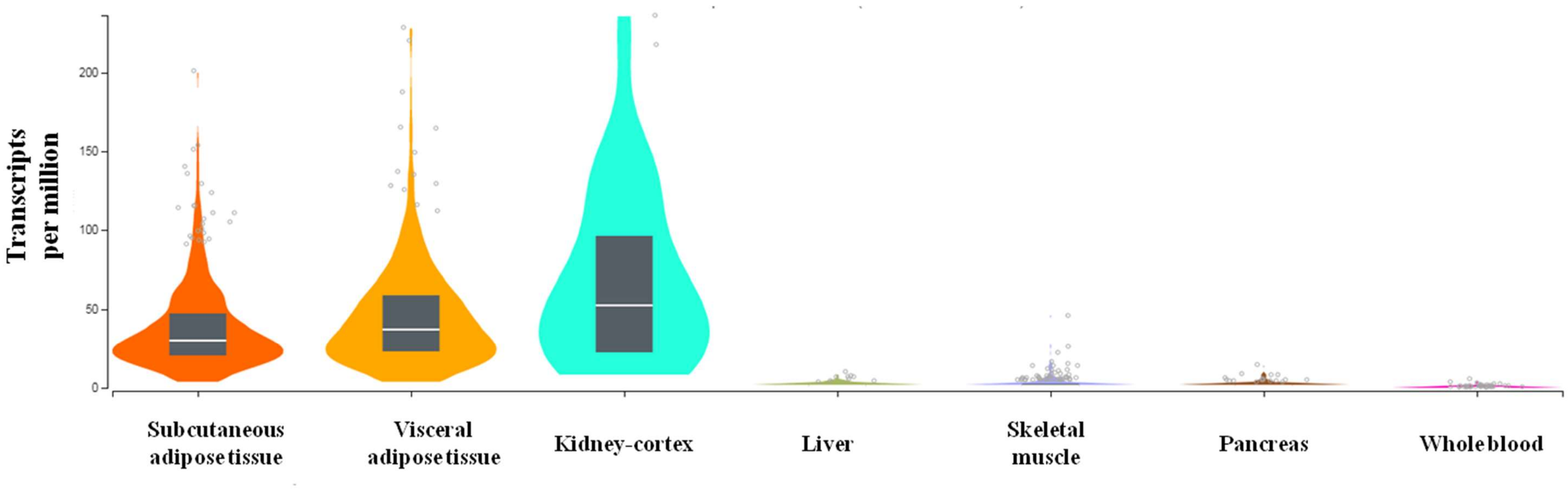

Data has been derived from GTEx portal [48]

Table S1: Characteristics of study population

Feature		Subjects without metabolic syndrome		Subjects with metabolic syndrome	
		Discovery phase	Replication phase	Discovery phase	Replication phase
Number (M/F)		562 (317/245)	2914 (1762/1152)	1596 (880/716)	5021 (2819/2202)
Age (years) Median (IQR)	Male	50 (45-59)	51 (44-60)	54 (45-62)	53 (46-62)
	Female	47 (42-55)	46 (41-55)	52 (45-61)	53 (45-61)
Waist circumference (cm) Median (IQR)	Male	85 (78-91)	89 (82-97)	86.36 (76.2-93)	96 (88.5-103)
	Female	81.28 (74-90)	83 (75.75-91)	88.9 (79.75-96.52)	92 (84-99)
Fasting glucose (mg/dl) Median (IQR)	Male	86.9 (79.5-93.4)	91 (83.8-99)	117 (90.35-156)	103.8 (90.6-141)
	Female	87.2 (81.2-92.8)	88.2 (81.8-95)	110 (88.35-158)	103.7 (90-148.4)
HDL-cholesterol (mg/dl) Median (IQR)	Male	43 (36.1-50.4)	45.59 (40-52)	36.1 (28.97-43.15)	38 (33-45.01)
	Female	53.21 (44.2-61.93)	52.5 (45.46-59.95)	42.4 (32.55-51)	43.3 (37.2-49.4)
Triglycerides (mg/dl) Median (IQR)	Male	94 (75.8-126.8)	99.25 (69.2-132)	124 (73.75-183.45)	140.2 (90.5-195.05)
	Female	89.8 (71.8-119.1)	86 (63.5-113.9)	136.7 (84.7-189.2)	131.7 (87.43-180.45)
Systolic blood pressure (mmHg) Median (IQR)	Male	120 (110-125)	120 (112-128)	130 (120-140)	131 (126-142)
	Female	120 (110-122)	118 (110-124)	130 (126-140)	130 (120-141)
Diastolic blood pressure (mmHg) Median (IQR)	Male	80 (70-80)	80 (74-82)	80 (78-90)	88 (80-90)
	Female	78 (70-80)	78 (70-80)	82 (80-90)	85.5 (80-90)

N: Number of subjects; IQR: Interquartile range

Table S2: Association status of known metabolic traits associated GWAS variants with MetS in replication phase in Indians ($p < 1x\ 10^{-3}$)

							Replication Phase		
**SNP**	**CHR**	**Base Position**	**Gene/Nearby Gene**	**SNP location**	**Alleles (Effect/Other)**	**MAF**	**N**	**p value**	**OR**
rs1800775#	16	56995236	*CETP*	Intergenic	C/A	0.38	4671	$3.48x10^{-9}$	1.29
rs3816117#	16	56996158	*CETP*	Intronic	A/G	0.39	4618	$7.71x10^{-9}$	1.29
rs7205804#	16	57004889	*CETP*	Intronic	G/A	0.49	4494	$1.58x10^{-6}$	1.23
rs1532624#	16	57005479	*CETP*	Intronic	A/C	0.49	4666	$5.57x10^{-6}$	0.83
rs3764261*	16	56993324	*CETP*	Intergenic	A/C	0.34	4673	$9.52x10^{-5}$	0.84
rs247617*	16	56990716	*CETP*	Intergenic	A/C	0.34	4665	$1.67x10^{-4}$	0.85
rs173539*	16	56988044	*CETP*	Intergenic	A/G	0.37	4656	$3.48x10^{-4}$	0.86
rs17782313@	18	57851097	*MC4R*	Intergenic	G/A	0.38	4666	$3.66x10^{-4}$	1.17
rs12970134$	18	57884750	*MC4R*	Intergenic	A/G	0.37	4649	$6.75x10^{-4}$	1.16
rs4128744#	8	19919655	*LPL*	Intergenic	A/G	0.09	4650	$8.82x10^{-4}$	0.79

Association analysis with compound MetS phenotype, adjusted for age and sex as covariates. SNP location is position of SNP in context of gene. CHR: Chromosome; MAF: Minor allele frequency; OR: Odds ratio. OR has been calculated with respect to the minor allele. * indicates earlier GWAS signals for compound MetS phenotype. # indicates earlier GWAS signals for lipid metabolism phenotypes. @ indicates earlier GWAS signals for obesity phenotypes. $ indicates earlier GWAS signal for obesity and type 2 diabetes. Earlier known GWAS signals for MetS and related metabolic traits were obtained from GWAS catalog [41].

Table S3: Reported eQTL and trait/disease associations of *CETP* region

eQTL associations of *CETP* loci							Reported associations with diseases	
**GWAS SNP**	**Alleles**	**Gene**	***P***	**Effect Size**	**Tissue**	**Source**	**Associated traits/diseases**	**Source**
rs1800775	C/A	*CETP*	4.30x10^{-8}	-0.24	Lung	[48]	Lipid metabolism, Coronary artery disease, Atrial Fibrillation, Chest pain/discomfort, Angina, Pulse rate, Weight, Waist-hip ratio, Hip circumference, Type 2 diabetes, Depression, Bipolar disorder, Anxiety, Alzheimer disease	[42]
			1.70x10^{-6}	-0.35	Cells-Tranformed fibroblasts			
			4.20 x10^{-6}	-0.4	Small intestine-Terminal illeum			
rs3816117	A/G		4.50 x10^{-8}	-0.23	Lung		Lipid metabolism, Coronary artery disease, Atrial Fibrillation, Angina, Heart rate, Weight, Waist-hip ratio, Cancer, Depression, Anxiety, Alzheimer disease, Alcohol dependency	
			7.90 x10^{-7}	-0.36	Cells-Tranformed fibroblasts			
			3.60 x10^{-6}	-0.4	Small intestine-Terminal illeum			
rs7205804	G/A		9.80 x10^{-8}	-0.39	Cells - Transformed fibroblasts		Lipid metabolism, Coronary artery disease, Atrial fibrillation, Pulse rate, Hypertension, Weight, Waist-hip ratio,Hip circumference, Depression, Anxiety, Alcohol dependency	
			1.30 x10^{-7}	-0.45	Small intestine-Terminal illeum			
			9.30 x10^{-6}	-0.2	Lung			
			1.20 x10^{-5}	-0.33	Stomach			
			2.80 x10^{-5}	-0.25	Esophagus - Mucosa			
rs1532624	C/A		9.80 x10^{-8}	-0.39	Cells - Transformed fibroblasts		Lipid metabolism, Coronary artery disease, Atrial fibrillation, Chest pain/discomfort, Pulse rate, Weight, Waist-hip ratio, Hip circumference, Depression, Anxiety, Alzheimer disease, Alcohol dependency, Rheumatoid arthritis	
			1.30 x10^{-7}	-0.45	Small intestine-Terminal illeum			
			6.10 x10^{-6}	-0.2	Lung			
			1.20 x10^{-5}	-0.33	Stomach			
			2.80 x10^{-5}	-0.25	Esophagus-Mucosa			
rs3764261	C/A		6.40 x10^{-11}	-0.3	Lung		Lipid metabolism, Coronary artery disease, Waist-hip ratio, Hip circumference, Pulse rate, Depression, Anxiety, Alcohol dependency, Insomnia, Rheumatoid arthritis,	
			1.20 x10^{-8}	-0.42	Colon-Transverse			
			2.80 x10^{-6}	-0.41	Small intestine-Terminal illeum			
			5.20 x10^{-6}	-0.29	Liver			
			1 x10^{-5}	-0.36	Stomach			
			1.20 x10^{-5}	-0.27	Esophagus -Mucosa			
			2 x10^{-5}	-0.34	Artery-Aorta			
rs247617	C/A		2.60 x10^{-11}	-0.31	Lung		Lipid metabolism, Coronary artery disease, Angina, Chest pain/discomfort, Pulse rate, Hypertension, Weight, Waist-hip ratio, Depression, Anxiety, Alcohol dependency, Insomnia, Schizophrenia, Cancer	
			1.50 x10^{-8}	-0.42	Colon-Transverse			
			5.80 x10^{-7}	-0.44	Small intestine-Terminal illeum			
			1.60 x10^{-6}	-0.31	Liver			
			5.50 x10^{-6}	-0.4	Pancreas			
			8.70 x10^{-6}	-0.37	Stomach			
			1.10 x10^{-5}	-0.28	Esophagus -Mucosa			
			1.10 x10^{-5}	-0.28	Adipose-Subcutaneous			
			2 x10^{-5}	-0.34	Artery-Aorta			
rs173539	G/A		1 x10^{-10}	-0.3	Lung		Lipid metabolism, Coronary artery disease, Angina, Chest	
			6.40 x10^{-9}	-0.43	Colon-Transverse			

			3.60 x10^{-6}	-0.41	Small intestine-Terminal illeum		pain/discomfort, Pulse rate, Hypertension, Body-mass index, Weight, Waist-hip ratio, Hip circumference, Waist circumference, Anxiety, Insomnia, Alcohol dependency, Cancer,	
			7.70 x10^{-6}	-0.4	Pancreas			
			9.70 x10^{-6}	-0.29	Liver			
			2.30 x10^{-5}	-0.26	Esophagus -Mucosa			

eQTL data for *CETP* SNPs has been retrieved from GTEx portal [48] and their earlier reported associations with trait/disease has been derived from GWAS atlas [42].

Table S4: Association status of known metabolic traits associated GWAS variants with MetS in replication phase in Indians (1x $10^{-3} < p > 0.05$)

							Replication Phase			
**SNP**	**CHR**	**Base Position**	**Gene/Nearby Gene**	**SNP location**	**Alleles (Effect/Other)**	**MAF**	**N**	**p value**	**OR**	**Associated MetS related trait in earlier GWAS studies**
rs2954029	8	126490972	*TRIB1*	Intergenic	*T/A*	0.35	4668	$1.56x10^{-3}$	0.87	Lipid metabolism phenotypes
rs9644568	8	19928582	*LPL*	Intergenic	*A/G*	0.09	4666	$1.60\ x10^{-3}$	0.8	Lipid metabolism phenotypes
rs328	8	19819724	*LPL*	Non-synonymous	*G/C*	0.08	4661	$1.96\ x10^{-3}$	0.79	Lipid metabolism phenotypes
rs964184*	11	116648917	*ZNF259*	Intergenic	*C/G*	0.2	4670	$2.61\ x10^{-3}$	1.17	Metabolic syndrome, Obesity, Lipid metabolism , Systolic and diastolic blood pressure, Glucose levels
rs10503669	8	19847690	*LPL*	Intergenic	*A/C*	0.08	4666	$2.65\ x10^{-3}$	0.79	Lipid metabolism phenotypes
rs7350481	11	116586283	*BUD13*	Intergenic	*A/G*	0.18	4389	$3.38\ x10^{-3}$	1.17	Lipid metabolism phenotypes
rs12678919	8	19844222	*LPL*	Intergenic	*G/A*	0.08	4543	$4.03\ x10^{-3}$	0.81	Lipid metabolism phenotypes
rs10096633	8	19830921	*LPL*	Intergenic	*A/G*	0.09	4671	$5.32\ x10^{-3}$	0.82	Lipid metabolism phenotypes
rs7561317	2	644953	*TMEM18*	Intergenic	*A/G*	0.14	4659	$5.56\ x10^{-3}$	0.85	Obesity
rs6511720	19	11202306	*LDLR*	Intronic	*A/C*	0.06	4666	$8.07\ x10^{-3}$	0.8	Lipid metabolism phenotypes
rs11242417	5	137599334	*GFRA3*	Intronic	*C/A*	0.09	4671	$8.56\ x10^{-3}$	0.82	Cytokine levels
rs8050136	16	53816275	*FTO*	Intronic	*A/C*	0.34	7911	$9.41\ x10^{-3}$	1.09	Obesity and Type 2 diabetes
rs4590817	10	63467553	*CABCOCO1*	Intronic	*C/G*	0.07	4671	$9.53\ x10^{-3}$	1.24	Systolic and diastolic blood pressure
rs1530440	10	63524591	*CABCOCO1*	Intronic	*A/G*	0.15	4656	0.01	1.16	Systolic and diastolic blood pressure
rs10808546	8	126495818	*TRIB1*	Intergenic	*A/G*	0.24	4632	0.01	0.88	Lipid metabolism phenotypes
rs3093059	1	157951759	*CRP*	Intergenic	*C/T*	0.09	3259	0.02	1.32	CRP levels
rs17367504	1	11862778	*MTHFR*	Intronic	*G/A*	0.18	4645	0.02	0.88	Systolic and diastolic blood pressure
rs3093077	1	157946259	*CRP*	Intergenic	*G/T*	0.11	3254	0.02	1.29	CRP levels
rs12594515	15	45985071	*SQOR*	Intergenic	*G/C*	0.48	4668	0.02	1.1	Obesity
rs7638110	3	138903985	*MRPS22*	Intergenic	*A/C*	0.01	4675	0.03	0.66	Obesity
rs2469997	8	120353267	*RF00421*	Intergenic	*C/G*	0.14	4665	0.04	1.13	Sytolic blood pressure and hypertension
rs6589567	11	116670676	*APOA5*	Intergenic	*A/C*	0.23	4675	0.04	1.11	Lipid metabolism phenotypes

Association analysis with compound MetS phenotype, adjusted for age and sex as covariates. SNP location is position of SNP in context of gene. MAF: Minor allele frequency; OR: Odds ratio. OR has been calculated with respect to the minor allele. * indicates earlier GWAS signals for compound MetS phenotype.

Table S5: Conditional analysis of *CETP* variants in Indians

		Replication phase		rs1800775		rs3816117		rs7205804		rs1532624		rs3764261		rs247617		rs173539	
**SNP**	**Gene**	**p value**	**OR**	**p value**	**OR**	**p value**	**OR**	**p value**	**OR**	**p value**	**OR**	**p value**	**OR**	**p value**	**OR**	**p value**	**OR**
rs1800775	*CETP*	$3.48 x10^{-9}$	1.29	NA	NA	0.36	1.13	$1.22 x10^{-3}$	1.23	$1.51 x10^{-4}$	1.27	$8.23 x10^{-6}$	1.26	$6.70 x10^{-6}$	1.26	$5.84 x10^{-6}$	1.26
rs3816117	*CETP*	$7.71 x10^{-9}$	1.29	0.28	1.15	NA	NA	$1.69 x10^{-3}$	1.24	$1.91 x10^{-4}$	1.28	$9.93 x10^{-6}$	1.26	$9.49 x10^{-6}$	1.26	$4.63 x10^{-6}$	1.28
rs7205804	*CETP*	$1.58 x10^{-6}$	1.23	0.35	1.06	0.49	1.05	NA	NA	0.05	1.47	$1.95 x10^{-3}$	1.19	$1.56 x10^{-3}$	1.19	$5.85 x10^{-4}$	1.19
rs1532624	*CETP*	$5.57 x10^{-6}$	0.83	0.69	0.98	0.94	0.99	0.35	1.2	NA	NA	0.01	0.87	$8.46 x10^{-3}$	0.86	$4.51 x10^{-3}$	0.86
rs3764261	*CETP*	$9.52 x10^{-5}$	0.84	0.43	0.96	0.54	0.97	0.34	0.95	0.17	0.92	NA	NA	NA	NA	0.19	0.86
rs247617	*CETP*	$1.67 x10^{-4}$	0.85	0.51	0.96	0.62	0.97	0.42	0.95	0.22	0.93	NA	NA	NA	NA	0.33	0.89
rs173539	*CETP*	$3.48 x10^{-4}$	0.86	0.63	0.98	0.83	0.99	0.42	0.96	0.18	0.93	0.89	0.98	0.68	0.95	NA	NA

Conditional analysis was carried in replication phase data for 7 *CETP* SNPs showing strong association with MetS in Indians. Logistic regression model adjusted for age, sex and SNPs genotypes was implemented for testing the associations.

Table S6: Haplotype association analysis of *CETP* locus for rs173539, rs247617, rs3764261, rs1800775, rs3816117, rs7205804 and rs1532624 respectively

Haplotype	Frequency cases	Frequency controls	OR	P	P*perm*
GCCCAGC	0.38	0.32	1.26	$7.97x10^{-8}$	$1x10^{-4}$
AAAAGAA	0.3	0.3427	0.84	$1.10\ x10^{-4}$	$6\ x10^{-4}$
GCCAGAA	0.15	0.167	0.88	0.03	0.15
AAAAGGC	0.01	0.02	0.786	0.19	0.74
GCCAGGC	0.07	0.07	0.96	0.63	0.99
ACCAGGC	0.02	0.02	0.942	0.69	0.99
GCCAAGC	0.02	0.02	0.937	0.72	0.99

Haplotype based association analysis was performed implementing sliding window approach in PLINK. Analysis was adjusted for age and sex. Association was regarded significant for $p<0.05$ at 10,000 permutation tests (P*perm*).

Table S7: Association analysis of novel imputed *SFRP1* variants with MetS in Indians

CHR	SNP	BP	N	OR	P	Gene	Location	Distance from index SNP (bp)
8	rs34836431	41181119	2103	0.39	$1.143x10^{-5}$	*SFRP1*	Intergenic	-9080
8	rs113128162	41237186	2157	0.46	$1.51x10^{-4}$	*SFRP1*	Intergenic	46987
8	rs57208963	41241486	2157	0.46	$1.51x10^{-4}$	*SFRP1*	Intergenic	51287
8	rs58477936	41255455	2158	0.46	$1.58x10^{-4}$	*SFRP1*	Intergenic	65256
8	rs73628732	41237699	2158	0.47	$1.93x10^{-4}$	*SFRP1*	Intergenic	47500
8	rs78026741	41244004	2158	0.47	$1.93x10^{-4}$	*SFRP1*	Intergenic	53805
8	rs58109926	41255254	2158	0.55	$2.30x10^{-4}$	*SFRP1*	Intergenic	65055
8	rs11986767	41184600	2123	0.68	$3.13x10^{-4}$	*SFRP1*	Intergenic	-5599
8	rs2197659	41193497	2144	0.68	$4.49x10^{-4}$	*SFRP1*	Intergenic	3298
8	rs76305295	41240287	2155	0.51	$1.15x10^{-3}$	*SFRP1*	Intergenic	50088
8	rs55900845	41205502	2144	0.56	$1.37x10^{-3}$	*SFRP1*	Intergenic	15303
8	rs2354571	41215331	2144	0.56	$1.37x10^{-3}$	*SFRP1*	Intergenic	25132
8	rs56309839	41185010	2121	0.71	$1.56x10^{-3}$	*SFRP1*	Intergenic	-5189
8	rs35367084	41234467	2134	0.52	$2.54x10^{-3}$	*SFRP1*	Intergenic	44268
8	rs16890462*	41190199	2156	1.29	$5.48x10^{-3}$	*SFRP1*	Intergenic	0

* marks index SNP
